# Multidimensional assessment of neuroendocrine and psychopathological profiles in maltreated youth

**DOI:** 10.1007/s00702-016-1509-6

**Published:** 2016-02-13

**Authors:** Vanessa B. Puetz, Jana Zweerings, Brigitte Dahmen, Caroline Ruf, Wolfgang Scharke, Beate Herpertz-Dahlmann, Kerstin Konrad

**Affiliations:** 1 Division of Psychology and Language Sciences, University College London, 26 Bedford Way, London, WC1H 0AP UK; 2 Child Neuropsychology Section, Department of Child and Adolescent Psychiatry, Psychotherapy and Psychosomatics, University Hospital RWTH Aachen, Aachen, Germany; 3 Department of Psychiatry, Psychotherapy and Psychosomatics, University Hospital RWTH Aachen, Aachen, Germany; 4 Department of Child and Adolescent Psychiatry, Psychotherapy and Psychosomatics, University Hospital RWTH Aachen, Aachen, Germany; 5 JARA Translational Brain Medicine, Aachen & Juelich, Germany

**Keywords:** Early-life stress (ELS), Early caregiver separation, Maltreatment, CBCL, Dimensional assessments, Cortisol, Aggression, Adoption, DSM

## Abstract

It has been debated whether children who have experienced early life stress (ELS), such as early caregiver separation show elevated risk for stress-related psychiatric disorders and a multi-symptom psychopathological profile that is not fully reflected in categorical assessments. In this study, we investigated dimensional measures of stress-related psychopathology in children in permanent out-of-home care, taking into account potential neuroendocrine interactions. In the current study, 25 children who had been placed in permanent out-of-home care before age 3 (years) and 26 controls (aged 10.6 ± 1.75 years) were investigated with categorical (DSM-IV) and dimensional assessments (CBCL) of psychopathology and diurnal salivary cortisol levels were assessed. Semi-structured interviews (K-DIPS) revealed no significant group differences in full-scale psychiatric diagnoses, whereas dimensional assessment (CBCL) revealed significant group differences in externalizing and total problem behaviours within the clinical range for children with ELS. Only children with ELS showed a combined symptom profile of clinical-range internalizing and externalizing problems. Lower morning cortisol values and subsequent flatter decline was found in subjects with ELS children compared to controls, showing group differences in diurnal cortisol secretion. Lower morning cortisol values were associated with more problem behaviour in the ELS group. Results show that ELS children exhibited increased psychopathological symptom severity and complexity associated with lower morning cortisol levels, which was not fully reflected in categorical assessments. This highlights the importance of incorporating dimensional assessments and neurobiological factors into psychopathological evaluations of children in out-of-home care in order to facilitate early identification of children at high risk for stress-related disorders.

## Introduction

Children who have experienced adversity in the form of maltreatment, neglect and subsequent caregiver separation have been exposed to severe early life stress (ELS), likely affecting neurobiological and neuroendocrine systems involved in stress-regulation. It has been shown that trauma-related stress is associated with the development of several psychiatric disorders such as major depressive disorder (MDD), post-traumatic stress disorder (PTSD), anxiety disorders and conduct disorder (CD) (Breslau et al. 2014; Cicchetti and Toth 1995; Heim and Nemeroff 2001; Klengel et al. 2014; Maercker et al. 2004; Mehta et al. 2011; Shields and Cicchetti 2001; see McCrory et al. 2010 for a review). Trauma-related stress early in life has also been associated with certain types of behaviour, such as impaired regulation of emotion, low self-efficacy and aggression, and has variable consequences at different developmental stages of the individual, thus resulting in age-related psychopathological symptoms (Van der Kolk 2005), e.g. regulatory and attachment disorders in infancy, conduct disorder at school age and personality disorders and substance abuse once maltreated individuals reach adolescence (Dahmen et al. 2012; Lo and Cheng 2007; Ouyang et al. 2008; Vaughn et al. 2007; Zeanah et al. 2004).

The psychopathology seen in children after early trauma-related stress typically manifests as a complex symptomatology, with high rates of comorbidity of about 47 % (Lawrence et al. 2006). Indeed, it has been shown that around three-quarters of children in care show levels of symptomatology that approach clinical populations with a multi-symptom pattern characterized by a broad range of mental problems rather than one diagnostic entity (Cecil et al. 2014; Lawrence et al. 2006; Oswald et al. 2010; Pears et al. 2008; Tarren-Sweeney 2008). It has been suggested that the increased symptom complexity across several psychopathological domains is a specific pattern of psychopathology that should be considered in its entirety when assessing individuals with early adverse experiences to facilitate early detection and intervention (Oswald et al. 2010; Tarren-Sweeney 2008; Van der Kolk et al. 2005).

In line with this, several studies have proposed that the current diagnostic classification systems (ICD-10 as well as the former DSM-IV) do not fully reflect the increased symptom complexity seen in subjects who experienced early adversity (Oswald et al. 2010; Van der Kolk et al. 2005). Because the boundaries between different diagnostic categories are blurred, cut-offs for categorical classifications might not be met despite complex and severe symptomatology, hindering early detection and intervention of individuals at high risk.

Considerable evidence has been presented that adverse postnatal experiences influence an infant’s ability to modulate its physiological response to stress, i.e. impacting the hypothalamic–pituitary–adrenal (HPA) axis (Gunnar and Donzella 2002; Sanchez 2006; Gunnar et al. 2001; Tarullo and Gunnar 2006). Increased risk for stress-related psychiatric disorders associated with early adversity could thus be mediated by biological factors. Research has shown that chronically elevated levels of adrenocorticotropic hormone (ACTH) and cortisol in response to repeated exposure to stress such as childhood maltreatment, can disturb the systems equilibrium by desensitizing glucocorticoid (CRF) receptors, increasing the risk for chronic HPA dysfunction later in life (Heim and Nemeroff 2001; Heim et al. 2000; Lupien et al. 2007; Sanchez 2006; Sapolsky et al. 2000).

Indeed, abnormal HPA-axis functioning has consistently been associated with early adversity and externalizing as well as internalizing symptoms (Cicchetti and Rogosch 2001a; Shirtcliff et al. 2005). This highlights the importance to address the interaction of neurobiological and psychosocial aspects in the development of complex psychopathological symptom clusters in children who experienced early adversity.

Animal studies with non-human primates have well established that maternal separation by itself is a powerful stressor that negatively impacts HPA-axis development. It has been demonstrated that peer- and nursery-reared monkeys show lower basal cortisol levels (Feng et al. 2011, although see Clarke 1993) than mother-reared monkeys, but elevated cortisol levels and greater adrenocortical responsiveness in response to a stressor, novelty or pharmacological challenge (Capitanio et al. 2005; Sanchez 2006). These results parallel studies in maternally separated rodents (Aisa et al. 2007; Plotsky et al. 2005).

Human research with maltreated children in foster care and adoption has extended the work from animal studies documenting HPA dysfunction after the experience of early life adversity. A majority of studies have identified lower basal cortisol levels in children in foster care and adoption (Bruce et al. 2009; Doom et al. 2014; Dozier et al. 2006; Kočovská et al. 2013; see Gunnar and Quevedo 2008 for a review). However, contrary findings have also been obtained. This might be due to several confounding factors, such as maltreatment type, severity and timing of maltreatment. For example, children with multiple abuse experiences showed higher cortisol levels than physically maltreated or neglected children (Cicchetti and Rogosch 2001a). In addition, the co-occurrence of psychiatric disorders might have contributed to heterogeneous findings. Previous findings in maltreated children with comorbid depression and internalizing symptoms are mixed, showing both elevated (Shirtcliff and Essex, 2008; Cicchetti and Rogosch 2001a) or decreased basal cortisol levels (Doane et al. 2013). Research with maltreated children with PTSD points towards significant elevations in basal cortisol (De Bellis et al. 1999) and in response to challenges (Badanes et al. 2011). While internalizing symptoms and affective disorders have been associated with both, hyper- and hypocortisolism (see Ruttle et al. 2011 for a discussion), a substantial amount of research has established an association between hypocortisolism and externalizing behaviours such as aggression and antisocial behaviour in adolescents (Hagan et al. 2014) and children (Jaffee et al. 2014; Murray-Close et al. 2008; van Goozen and Fairchild 2006, see Alink et al. 2008 for a review).

The current study aimed to investigate (1) the psychopathological symptom patterns in a sample of children with a history of childhood maltreatment and subsequent early caregiver separation (early life stress group; ELS) who were removed from the adverse environment before their 3rd year of life using categorical and dimensional psychiatric assessments, and (2) to investigate diurnal basal cortisol levels and potential associations with psychopathology, and (3) to investigate risk and protective factors (i.e. current caregiver relationship, time spent in foster/adoptive family) as potential predictors of psychopathology after ELS. Based on previous research, we hypothesized that children with ELS show a multi-symptom psychopathological profile as well as altered diurnal cortisol levels.

## Materials and methods

### Participants

The sample consisted of 25 youth who have experienced severe early life stress in the form of childhood maltreatment and were subsequently permanently separated from their biological parents and placed into long-term foster or adoptive families. The youth with ELS (13 male and 12 female) were between 8 and 14 years old (mean age 10.6 years ± 1.75 years) at the time of assessment, and ethnicity was distributed as follows: 53.8 % (*n* = 14) Caucasian, 30.8 (*n* = 8) Asian and 11.5 % (*n* = 3) Hispanic. Because the first 3 years of life are considered to be a sensitive period for attachment formation (Bowlby 1969), we included only youth in the study who were separated from their mothers between birth and their third year of life (mean age of separation, 1.59 ± 1.05 years). Importantly, to minimize the likelihood that youth differed in exposure to present socioeconomic or psychological stressors related to placement instability, we included only children and adolescents in permanent placements such as adoption (*n* = 16) or permanent foster care (*n* = 9). Children’s medical records were checked for written evidence for developmental delay, growth retardation, physical or sexual abuse, neglect and physical maltreatment. In addition, a semi-structured biographical interview (Groh 2010) was conducted with the caregivers to obtain information about the circumstances of separation. This interview covers in particular the reasons for separation from the birth parents, the number of transitory placements that the child experienced before placement in the permanent family and the kind of placement.

The primary form of maltreatment and main reason for separation from their biological parents was emotional and physical neglect (64 %, *n* = 16), followed by abandonment (24 %; *n* = 6), physical abuse (8 %, *n* = 2) and one child entered care due to witnessing severe domestic violence (4 %; *n* = 1). Of those 25 youths, 11 (44 %) were immediately placed into permanent foster or adoptive families without secondary placements, and 14 experienced intermediate secondary placements lasting up to a maximum of 12 months (range total number of placements, 1–4) before their third year of life. At the time of assessment youths lived for an average of 9.14 ± 2.27 years (range 4.50–13.48) in their permanent families. None of the ELS youths lived in the care of kin. Nine children in the ELS group had a non-pervasive developmental disorder in the past and all but one (F80.9 Developmental disorder of speech and language) were presently remitted. Additionally, a comparison group of 26 control youths who grew up with their biological parents and had never been in contact with social services were included in the study (12 male, 14 female; mean age 10.38 ± 1.67 years; 85 % Caucasian, 15 % mixed race). The groups did not differ significantly in terms of age [*t*(49) = −.45, *p* = .66)], sex [*χ*^2^(1) = .07, *p* = .78], IQ [(*t*(49) = 1.03, *p* = .18)] or parental socioeconomic status [*U*(49) = 238.5, *Z* = −1.32, *p* = .19]. However, the groups did differ significantly on nationality [*χ*^2^(3) = 22.56, *p* < .001]. All 51 youths met the following study inclusion criteria: (a) IQ equal to or greater than 85; (b) no current pharmacological treatment (*n* = 1 control and *n* = 2 ELS youths discontinued short-acting methylphenidate treatment at least 48 h prior to saliva collection), and (c) no present or past neurological disorder or brain injury.

This study has been conducted in accordance with the Declaration of Helsinki and its later amendments and was approved by the local ethics committee at Rheinisch-Westfälische Technische Hochschule Aachen (RWTH Aachen University). All participants and their legal caregivers gave written informed assent and consent to participate in this study.

#### Assessments

For all participants, IQ was assessed using the Wechsler Abbreviated Intelligence Scales (Wechsler 1999). SES was operationalized through measures characterizing the highest parental educational levels within the foster/adoptive (for the ELS group) or biological family (in the control group).

### Assessment of psychopathology

Diagnostic classification according to DSM-IV criteria (APA 1994) was assessed with a semi-structured diagnostic interview conducted with the youths and their caregivers separately (K-DIPS; Unnewehr et al. 1995). The frequency of symptoms is rated on a 4-point Likert scale from 0 = never/seldom to 3 = very often (scores of two or higher indicating the presence of symptomatology). Furthermore, the intensity of the symptoms (i.e. degree of distress caused by the symptoms and impairment of daily life) is also rated on a 4-point Likert scale (0 = not at all and 3 = very strong). The diagnostic category with the highest general severity rating, as obtained through both, frequency and intensity scores, is rated for the primary diagnosis. If children also meet criteria for other DSM-IV diagnoses, those are considered secondary. The K-DIPS is a valid instrument to assess psychological disorders and has a moderate to very good test–retest reliability (child version 0.48–0.88; parent version 0.85–0.94; Adornetto, In-Albon and Schneider 2008). A dimensional measure of symptom severity and behavioural problems was obtained via the parent version of the Child-Behaviour Checklist for youth between 4 and 18 years of age (CBCL; Achenbach 1991). The CBCL was scored in a standardized way, yielding scores per subscale as well as two scores for internalizing and externalizing behaviour and a total score (Achenbach 1991). In order to assess impulsivity, venturesomeness and empathy, the German version of the Impulsiveness–Venturesomeness–Empathy Questionnaire was used (IVE; Eysenck and Eysenck 1991; Stadler et al. 2004). This measure yields one score for each subscale (i. Impulsiveness; ii. Venturesomeness; iii. Empathy). To obtain a measure of the quality of the caregiver–youth relationship from the youth’s perspective, we administered the Parental-Representation-Screening-Questionnaire (PSRQ; German tite: Elternbildfragebogen für Kinder und Jugendliche, EBF-KJ; Titze et al. 2005). The PSRQ is a self-report questionnaire for children consisting of 72 items, measuring stress factors as well as positive resources within the relationship (“emotional burden by the parents”, “fears / overprotection”, “conflict”, “hostility / indifference”, “aid for the parents”, “punishment”, “freedom of decision” and “support from the parents”). The resulting total score describes the quality of the parent–child relationship. The PSRQ total index has been shown to have good internal consistency (Cronbach’s alpha = 0.75) and retest-reliability (0.84; Titze et al. 2010). Between-group analyses with non-parametric Mann–Whitney *U* tests revealed no significant differences between the quality of the relationship with the caregiver for youths raised by their biological parents and ELS youths [*U*(48) = 228.0; *Z* = −1.24, *p* = .22]. On average, youths in both groups indicated a high relationship-quality with their caregivers [mean *T* values controls 71.17 ± 7.79; ELS 68.08 ± 8.63, corresponding to the 96th–98th percentile]. The number of close friends, as evidenced by parental ratings, did not differ significantly between the ELS and control group [*χ*^2^(2) = .80 *p* = .62], indicating that both groups were comparable in terms of current social integration.

Finally, because foster care populations present with an increased risk for foetal alcohol syndrome (FAS) of up to 15 % above the general population (Astley and Kinzel 2002) and FAS potentially impacts on HPA-axis functioning (Schneider et al. 2002; Zhang et al. 2005), we included the FAS Facial Photographic Screening Tool (Version 1.0.0) to measure the magnitude of the FAS facial phenotype expression in the ELS group. The software revealed no facial features indicative of FAS in 21 ELS (84 %) youths and identified 4 (16 %) youths with mild FAS facial features. No participant presented with clear FAS facial features. FAS features were subsequently controlled in all data analyses. Demographic and clinical characteristics for both groups separately are shown in Table 1.

**Table 1:** Demographic characteristics for the control and ELS children in the sample

	ELS children *N* = 25 (*N* = 17)^a^		Controls *N* = 26 (*N* = 23)^a^		*p*
	Mean	SD	Mean	SD	
Age	10.6 (10.88)	1.75 (1.65)	10. 38 (10.30)	1.67 (1.74)	0.66 (0.30)
IQ	100.52 (101.94)	10. 66 (11.55)	104.34 (103.30)	9.19 (8.96)	0.18 (0.68)
SES	2.5 (2.46)	0.83 (0.83)	2.84 (2.81)	0.94 (0.96)	0.19 (0.26)

	*n*	%	*n*	%	*p*
Gender					
Boys	13 (12)	52 (71)	12 (9)	46 (39)	0.78 (<0.05)
Girls	12 (5)	48 (29)	14 (14)	54 (61)	
Ethnicity					
Caucasian	14 (7)	56 (41)	22 (19)	85 (83)	0.001 (<0.001)
Mixed	0 (0)	0 (0)	4 (4)	15 (17)	
Asian	8 (8)	32 (47)			
Hispanic	3 (2)	12 (12)			
Past developmental delay^1^	9 (8)	36 (47)	0	0	0.002 (<0.001)
Stimulants	2 (2)	8 (8)	1 (1)	4 (4)	0.49 (0.17)

^a^Numbers in brackets are calculated based on the sub-sample for which valid salivary cortisol data were available, i.e. *n* = 23 controls and *n* = 17 ELS children. ^1^Information based on parental or paediatric documentation: motor delay (*n* = 3), language delay (*n* = 6) within the first 3 years of life, all remitted

### Salivary cortisol assessment

Cortisol was collected using the Sarstedt Salivette device (Sarstedt Inc., Rommelsdorf, Germany). Caregivers were instructed to place the Salivette cotton rolls into the child’s mouth and ensure that the cotton roll was chewed for at least 1 min until sufficient saturation with saliva before placing them back into the plastic vials. Caregivers were instructed to note the exact time and date on the vials and place samples in the refrigerator immediately after collection and either mail them to our lab via post or bring them to our lab within 48 h after collection (following the procedure of Kočovská et al. 2013). After unfreezing, all saliva samples were centrifuged at 2000×*g* for 2 min to separate the saliva from the swab and were afterwards brought to the central laboratory of the University Hospital Aachen. Samples were assayed by electrochemiluminescence-immunoassay (ECLIA; Cobas e601). The lower detection limit of the assay is 0.5 nmol/l and the higher detection limit is 1750 nmol/l. To minimize within-subject variability within the sample, all samples belonging to one participant were assayed in the same batch.

Saliva was collected by the caregivers three times a day (morning: between 7 and 9 a.m. and 30 min after awakening, noon: 30 min before lunch, and evening: 30 min before going to sleep) on two consecutive days to assess the diurnal cortisol pattern of the participants. We chose to sample the cortisol 30 min post-awakening over immediately after awakening in order to capture the peak daily cortisol production as cortisol rises up to 60 % within 30 min after awakening (Schmidt-Reinwald et al. 1999).

To ensure adherence to the sampling protocol, caregivers were asked to complete a questionnaire in which they had to record the exact time and date of collection and indicate various issues known to potentially interfere with cortisol assessment, such as the occurrence of any special or stressful events on the days of collection, sleeping behaviours, certain medications (e.g. steroidal asthma inhalers) and non-adherence to protocol due to eating or brushing teeth shortly before collection. Samples were excluded wherever non-adherence to the protocol was indicated (see below).

### Salivary cortisol analyses

#### Daytime values

Since correlational analyses revealed a high association between the cortisol measurements of both days, morning [*r*_morning_ = .55, *p* < .001, *n* = 30], noon [*rs*_noon_ = .75, *n* = 34] and evening [*rs*_evening_ = .77, *p* < .001, *n* = 39] cortisol data was averaged over the 2 days separately for the three time points of collection to maximize reliability of the measurements. Data points that were 2.5 standard deviations above the sample mean (*M*_Morning_ = 12.67 ± 5.72; *M*_Noon_ = 3.77 ± 3.25, *M*_Evening_ = 3.31 ± 3.38) were replaced with the next highest value below 2.5 standard deviations above the sample mean (Tukey 1977). This resulted in one morning, one noon and three evening replacements. Morning values were normally distributed and non-parametric tests were used when non-normality was indicated. Out of the 51 youths who participated in the study, 4 dropped out of the analyses because of lacking or insufficient saliva collection. Because sampling time in the morning has been shown to substantially influence cortisol levels (Adam and Kumari 2009; Adam and Gunnar 2001; Kudielka et al. 2003) 7 participants were excluded because of non-adherence to the sampling instructions, i.e. saliva sampling before 7:00 or after 9:00 am or missing documentation of collection time. Sampling time did not differ between the groups for any of the three sampling time points [morning: *t*(38) = −1.23, *p* = .23; noon: *t*(38) = .54, *p* = .59; evening: *t*(38) = −.43, *p* = .67]. The final number of valid datasets (case-wise) used for the analyses of the cortisol values was 17 ELS youths and 23 controls (listwise 16 vs. 20). The groups did not differ significantly in terms of age [*t*(38) = −1.06, *p* = .30)], IQ [*t*(38) = .41, *p* = .68] or socioeconomic status [*t*(35) = 1.15, *p* = .26]. However, the groups did differ significantly on gender distribution [*χ*^2^(1) = 3.88, *p* = .49] and nationality [*χ*^2^(3) = 19.07, *p* < .01]. Effects of sex and nationality were therefore controlled in exploratory cortisol pre-analyses.

## Results

### Categorical assessment of psychopathology

The diagnostic classification according to DSM-IV criteria (APA 1994) revealed 9 youths in the ELS group (36 %) that met the diagnostic criteria for either ADHD (DSM-IV 314.0, *n* = 6), conduct disorder (DSM-IV 312.81, *n* = 1); dyslexia/dyscalculia (DSM-IV 315.0, *n* = 1) or enuresis (DSM-IV 307.6, *n* = 1). Five children in the control group (19 %) met the criteria for either ADHD (*n* = 1) or dyslexia/dyscalculia (*n* = 4; see Table 2). Analysis of the diagnostic classifications revealed no significant difference in the frequency distribution of full-scale psychiatric diagnoses versus no diagnoses according to DSM-IV across the ELS and control group (*χ*^2^(1) = 1.37, *p* < .34). A statistical frequency distribution of distinct diagnostic categories was not conducted because of the small cell frequency (expected cell-count less than 5). Note, however, that none of the participants met the diagnostic criteria for affective disorders and only one participant met the formal diagnostic criteria for an externalizing disorder (conduct disorder).

**Table 2:** Mean *T* values separated by group for the CBCL (Achenbach 1991), the IVE (Eysenck and Eysenck 1991; Stadler et al. 2004) and frequency distribution for full-scale DSM-diagnosis as measured with the K-DIPS

Measures	ELS		Controls		*p* value
	Mean	SD	Mean	SD	
CBCL					
Socially withdrawn	60.92	9.06	53.48	4.69	<.001
Anxious/depressed	60.03	9.36	53.28	4.81	.001
Social problems	61.19	12.25	54.08	6.84	.007
Schizoid	61.73	9.31	52.88	5.01	<.001
Physical complaints	55.5	7.07	56.24	7.96	.36
Attention	63.5	7.85	63.5	6.78	.001
Dissocial	60.42	7.76	54.08	7.11	.002
Aggressive	67.03	11.33	54.6	7.01	<.001
Internalizing	60.15	8.35	52.04	7.66	.001
Externalizing	64.5	9.83	51.72	9.26	<.001
Total	63.73	8.87	51.96	8.58	<.001

	Mean	SD	Mean	SD	*p* value
IVE					
Impulsivity	57.36	11.1	49.07	11.4	.005
Venturesomeness	49.44	9.46	48.5	7.03	.34
Empathy	51.44	11.58	53.9231	9.24	.201

	*n*	%	*n*	%	*p* value
K-DIPS					
DSM-criteria met	9	36	5	19	.345
DSM-criteria not met	16	64	21	81	

### Dimensional assessment of psychopathology

Analyses of the CBCL (Achenbach 1991) revealed a significant group difference in the total CBCL score (*t(49)* = −4.81, *p* < .001) and all scales measuring problem behaviour except for somatic problems after Bonferroni correction (*t(49)* = −.35, *p* = .72). Inspection of mean *T* values showed that, as expected, ELS youths displayed significantly higher values on all subscales except for somatic problems (see Table 2 for mean values per subscale).

**Fig. 1:**
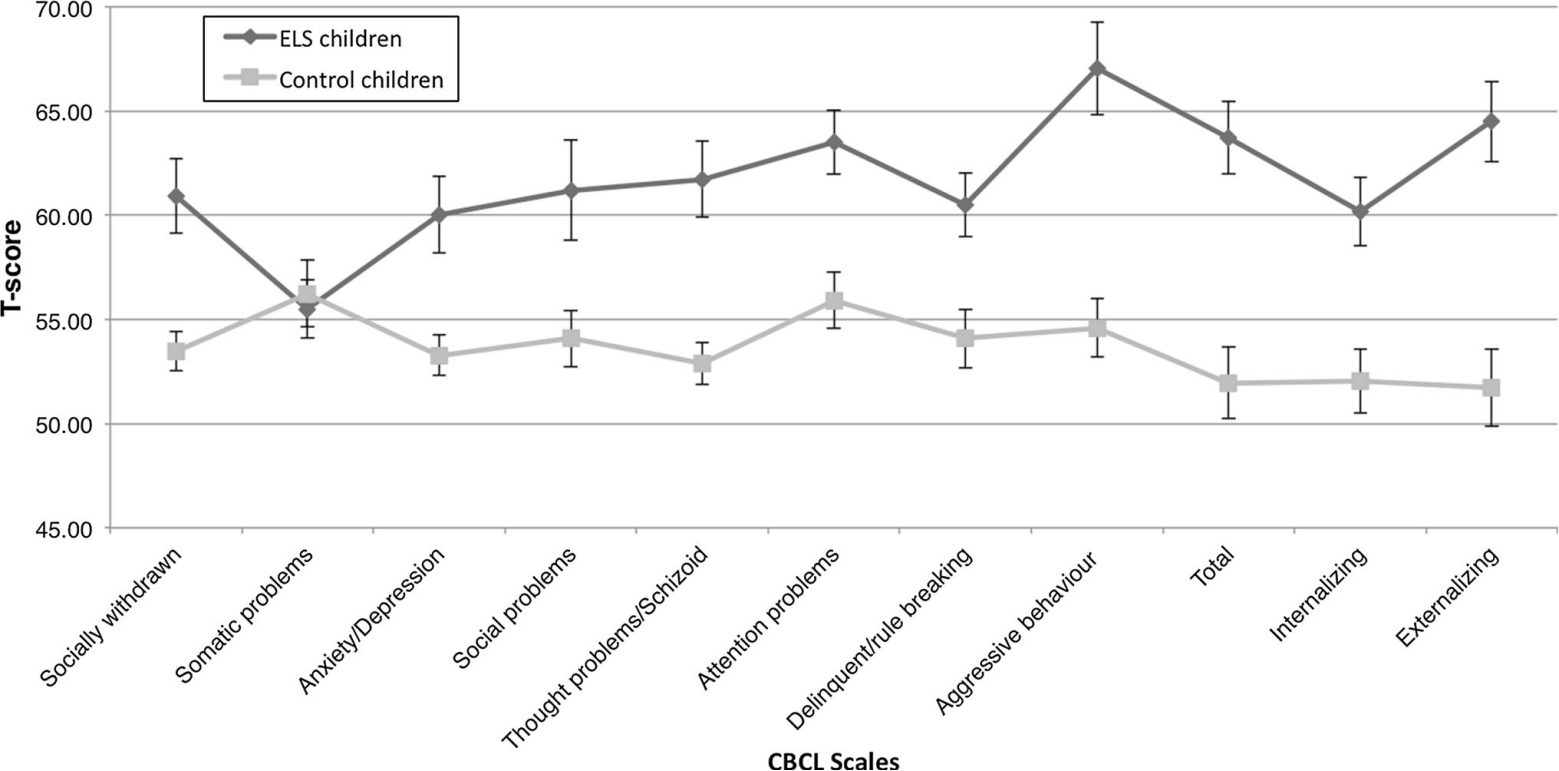
Distribution of *T* values for each of the CBCL syndrome scales per group (ELS group *n* = 25; controls *n* = 26). Note that the value for aggressive behaviour for ELS youths in the sample fell within the borderline clinical range and the average total value fell within the clinical range (syndrome scale values <65 fall within the normal range, values between 65 and 69 fall within the borderline range and values >69 fall within the clinical range. Total scale values <60 fall within the normal range, values between 60 and 63 fall within the borderline range and values >63 fall within the clinical range)

In the group of ELS youths, overall internalizing problems were in the borderline clinical range (>60 to <63), whereas externalizing and total problems behaviours were within the clinical range (>63). Further inspection of the externalizing syndrome scales showed that ELS youths obtained the highest score on aggressive behaviour scale, which was within the borderline clinical range (65–69). Within the control group, mean values for the whole group for all syndrome and total scales fell within the normal range (see Fig. 1).

**Fig. 2:**
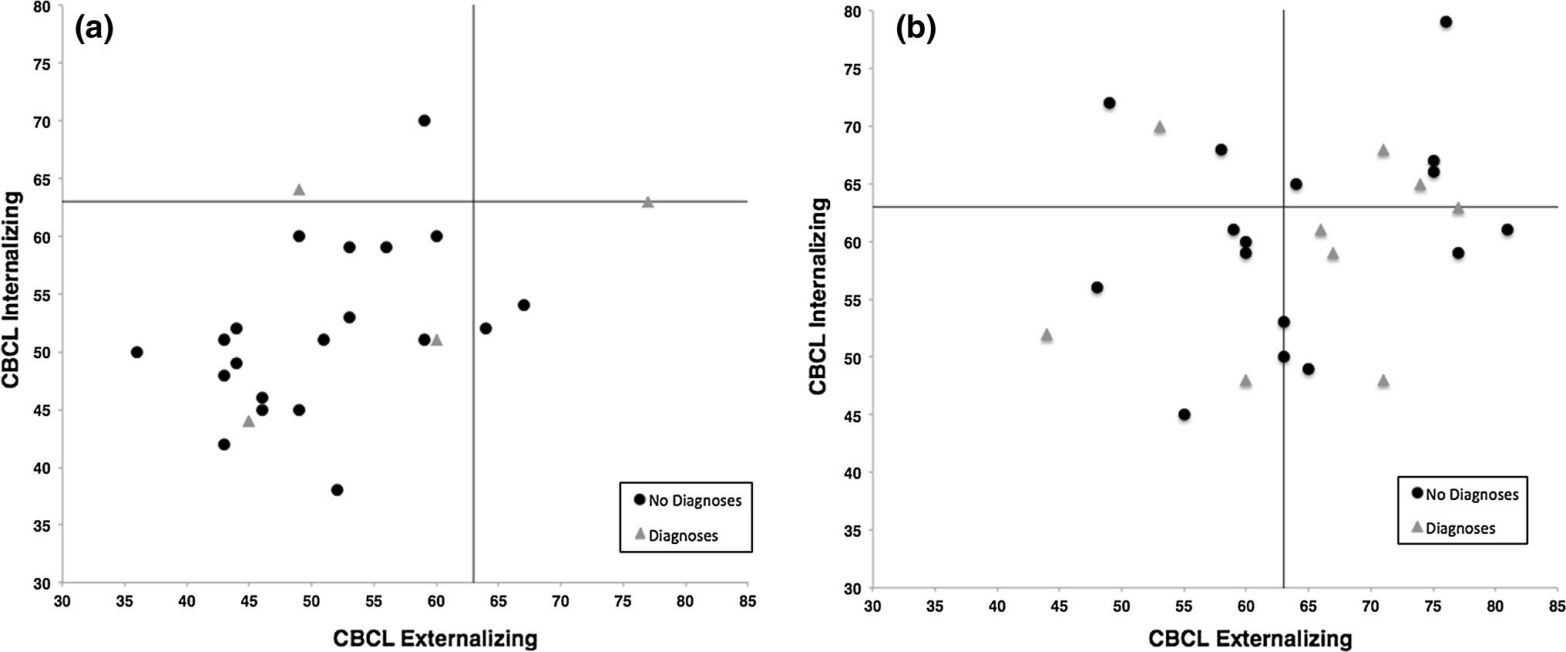
Scatterplots depicting the association between internalizing and externalizing scores for **a** control children and **b** ELS youths with and without categorical DSM-IV diagnoses. *Vertical* and *horizontal lines* mark the clinical range cut-off (*T* = 63). Note that none of the control youths (**a**) shows a combined symptom profile of externalizing and internalizing problems within the clinical range which is evident in *n* = 6 ELS youths (**b**)

Interestingly, while CBCL internalizing and externalizing symptoms were significantly associated with each other in the control group (*r* = .50, *p* = .01), this was not the case for youths with ELS (*r* = .25, *p* = .23). Considering the individual values in the control group, five out of 26 children (19 %) fell within the clinical range of either internalizing or externalizing symptoms (*n* = 2 fulfilling DSM-IV criteria for a mental disorder); however, none of the control participants had a combined symptom profile of internalizing and externalizing symptoms (see Fig. 2). In the ELS group, 16 out of 25 children (64 %) fell in the clinical range of either externalizing or internalizing behaviour problems, and six of them (25 %) showed a combined symptom profile (of which only *n* = 2 fulfilling DSM-IV criteria for a mental disorder).

Analysis of the IVE (Eysenck and Eysenck 1991; Stadler et al. 2004) showed that while ELS youths exhibited significantly more impulsive behaviours than control youths in our sample [*t(49)* = −2.62, *p* = .005]; there were no differences in venturesomeness or empathy (both *p* > .20) (Table 2).

## Group differences in cortisol daytime levels

Since there are no universally valid cut-off values for cortisol data in children and values vary from study to study because of different assaying techniques and sampling procedures, we informally compared our salivary cortisol values with values previously obtained in control- and maltreated paediatric samples. Comparison of our data with the values reported by (Bruce et al. 2009), and normative values obtained from (Tollenaar et al. 2010) revealed that our data lay between the 5th and 95th percentile of the normative sample and was within physiological limits. Mean cortisol values for the three collection timepoints are presented in Table 3.

**Table 3:** Mean cortisol values in the morning, at noon and in the evening for both groups

	EL	S (*n* = 17*)*	Controls	(*n* = 23)
	Mean	SD	Mean	SD
Morning	10.8	5.27	14.03	4.95
Noon	3.45	3.21	3.48	1.7
Evening	2.94	4.01	2.91	2.83

All values are in nmol/l. *n* is listwise

### Explorative analyses

In order to rule out potential confounding effects, univariate ANOVAs were carried out to investigate the influence of age, gender and nationality on our dependent variable. No significant relationship between morning, noon or evening cortisol values and age [*F*_Morning_(1,35) = .24, *F*_Noon_(1,36) = 1.26 *F*_Evening_(1,37) = 2.56 all *Ps* > .05], gender [*F*_Morning_(1,35) = 3.13, *F*_Noon_(1,36) = 1.24, *F*_Evening_(1,37) = 3.98; all *P*s > .05] or nationality [*F*_Morning_(3,35) = 1.34, *F*_Noon_(3,35) = .66, *F*_Evening_(3,35) = .86; all *P*s > .05] were found.

Because time of sampling has been shown to influence cortisol levels (Kudielka et al. 2003), we investigated time of sampling as a possible confound. A significant relationship between time of sampling and morning and evening values was found (*r*_Morning_ = −.39, *p* = .02, *n* = 36; *rs*_Evening_ = .37, *p* = .025) while noon cortisol values were not associated with sampling time (all *rs*_Noon_ = −.10, *p* = .55). Importantly, sampling time did not differ between the groups for any of the three sampling time points [morning: *t*(38) = −1.23, *p* = .23; noon: *t*(38) = .54, *p* = .59; evening: *t*(38) = −.43, *p* = .67].

**Fig. 3:**
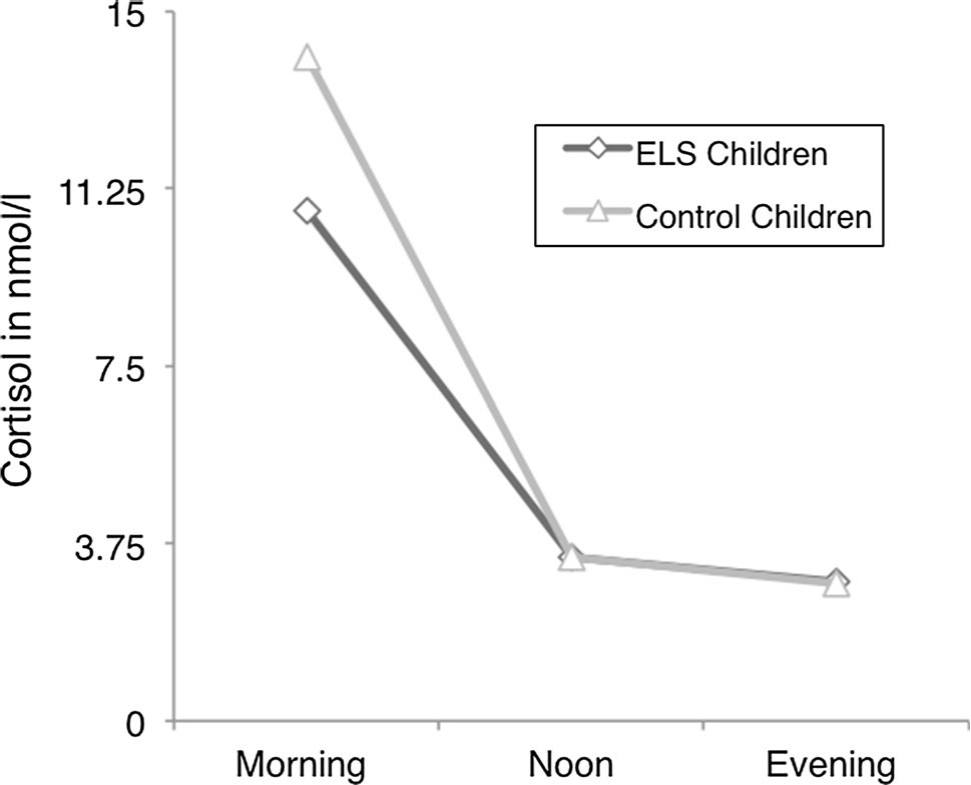
Cortisol release pattern over the three measurement points for ELS and control youths. The morning cortisol release is significantly lower in ELS youths than controls

In order to investigate group differences in the amount of cortisol secretion over the three collection times of the day and differences in secretion pattern, a repeated measures analysis of variance (ANOVA) was carried out with time-point as within-subject factor and group as between-subject factor. A significant interaction between group and cortisol level at the different time points was found, indicating that the level of diurnal cortisol secretion differed between ELS youths and controls [*F*_GG_(1.65, 54.65) = 3.55, *p* = .04; see Fig. 1]. Post hoc analyses indicated that the difference in cortisol secretion pattern was due to significantly lower morning cortisol in ELS youths as compared to controls [*F*(1,33) = 4.57, *p* = .04], in the absence of significant secretion differences at noon or in the evening [*F*(1, 33) = .12, *p* = .73; see Fig. 3)]. Additional analyses were carried out to investigate differences in the diurnal slope between the ELS group and controls, using the diurnal slopes derived from the average morning, noon and evening values. Following the protocol outlined by DeSantis et al. (2007) and Stone et al. (2001), we calculated each participant's individual slope coefficients by regressing their cortisol values on the sampling times for morning, noon and evening to obtain estimates of individual diurnal profiles. Slopes were normally distributed [KG-S(37) = .067, *p* = .20]. As expected, significant group differences emerged in the diurnal cortisol slopes (*t*(34) = −2.21, *p* = .034), with ELS children showing smaller coefficients and thus, significantly flatter slopes (mean ELS: −14.83 ± 9.67; mean controls: −21.61 ± 8.40).

Despite significant group differences in the level of cortisol release (i.e. lower morning values) and slopes during the day, both groups showed the physiologically expected diurnal decrease in cortisol after the peak in the morning (Fig. 3).

In order to control for the effects of behavioural disorders on between-group differences in daily cortisol excretion, CBCL *T*-scores for either internalizing, externalizing or total problem behaviour were entered as covariates into the model, however, none of them showed a significant effect (all *P*s > .15). Correlational analyses were carried out between those cortisol parameters that differed between children with ELS and controls (morning cortisol levels, cortisol diurnal slopes) and CBCL externalizing, internalizing and total problem behaviour, separately per group. Alpha-level was adjusted group-wise (Bonferroni corrected at *p* < .008) to correct for multiple comparisons. Correlational analyses separately per group revealed a significant negative relationship in the ELS group between participant’s cortisol production in the morning and the total score of the CBCL (*r* = −.65, *p* = .007), indicating that lower cortisol values in the morning were associated with higher total scores on the CBCL only in ELS cases. Cortisol diurnal slopes did not correlate significantly with the CBCL total, externalizing or internalizing problem scales (*P*s > .09). No significant correlations emerged between CBCL scores and morning levels or diurnal slopes in the control group (all *P*s > .17).

### Discussion

In the present study, we examined mental health and daily basal cortisol production in a well-characterized sample of maltreated youths in permanent foster care and adoption that were comparable to non-maltreated youths in terms of social integration and current caregiver-child relationship. Our results showed that even after several years of living in a stable environment ELS youths exhibited deviant patterns of diurnal cortisol secretion, as well as increased symptom severity and multi-symptom complexity that was not fully captured by categorical assessments (i.e. DSM-IV). Moreover, abnormal HPA functioning in the form of lower morning cortisol secretion in youths with ELS was associated with increased symptom severity; however, could not be predicted by risk or protective factors.

The finding of increased symptom severity and multi-symptom profile of elevated internalizing and externalizing problem behaviour in our sample of ELS youths is in line with previous research showing that youths who experience early life adversity score within clinical ranges on a broad range of syndrome scales (Cecil et al. 2014; Oswald et al. 2010; Tarren-Sweeney 2008; Van der Kolk et al. 2005). In the present sample, DSM-IV criteria did not adequately reflect the psychopathological profile, possibly because full-scale psychiatric disorders had not yet emerged. However, dimensional assessments revealed total problem behaviours, specifically externalizing behaviour, within the clinical range. Importantly, dimensional assessment not only revealed increased symptom severity in ELS youths, but also a multi-symptom profile of concurrently elevated internalizing and externalizing behaviour that was uniquely present in about one-quarter of youths with ELS. This finding supports the notion that classification with a DSM-IV interview did not fully capture the complex nature of symptoms displayed by ELS children and should standardly be supplemented with syndrome scales to facilitate adjustment of therapeutic needs in maltreated children and adolescents. This approach could further facilitate the identification of a specific pattern of symptomatology and thus benefit the identification of high-risk individuals in clinical practice.

The finding of significantly reduced basal cortisol levels in the ELS group in our study even after approximately 10 years within a stable family environment replicates previous findings in samples of children living foster care and adoption (Bruce et al. 2009; Doom et al. 2014; Dozier et al. 2006; Kočovská et al. 2013). This provides further evidence for the pervasive influence of early adversity on neuroendocrine functioning, which has been shown to persist into emerging adulthood (Hagan et al. 2014). Although blunted daily cortisol patterns after chronic exposure to stress have initially challenged prevailing theories on stress, it is now well established that hypocortisolism likely reflects processes within the organism to adjust to the stressful environment (allostatic adjustment) which is considered to be adaptive in the short-term, but poses a major threat to healthy development (Heim et al. 2000; Badanes et al. 2011; Gunnar and Vazquez 2001) at the behavioural (Lupien et al. 2007; Shoal et al. 2003) and neural level (McEwen 2000). In the light of the allostatic load model (McEwen 1998), it has been proposed that basal HPA suppression is the consequence of prolonged allostatic adjustments in which the organism tries to adapt to the adverse and stressful environment by creating a state opposite to the one that brought the change, ultimately to restore homeostasis to the organism. On the neural level, allostatic load is thought to be reflected by a down-regulation of corticotrophin releasing factor (CRF) receptors in response to elevated levels of glucocorticoids over prolonged periods of chronic stress or trauma in the form of early adversity or caregiver separation (Heim et al. 2000). Importantly, adding to previous research in the field demonstrating abnormal neuroendocrine functioning in youths with a history of early adversity, we here demonstrated that basal HPA suppression remained significant after controlling for CBCL problem behaviour, foetal alcohol syndrome features and current social integration with peers and caregivers. This finding of pervasive neuroendocrine dysregulation and clinical levels of aggression despite ‘best-case’ environmental conditions (i.e. good present caregiver–child relationship; stable environment) could bear important implications for this group of youths and their families, as abnormally low cortisol values have previously been shown to predict future exhibition of aggressive, delinquent and antisocial behaviours (Lorber 2004; Oosterlaan et al. 2005; Raine 1996; Shoal et al. 2003).

In line with this, lower morning cortisol values in the ELS group were significantly associated with increased levels of behavioural problems as measured by the CBCL, paralleling previous associations between reduced cortisol values and problem behaviour in youths with and without early life adversity (Alink et al. 2008; Hagan et al. 2014; Jaffee et al. 2014; Murray-Close et al. 2008; van Goozen and Fairchild 2006).

Considering the small sample size, caution needs to be exercised when interpreting the results. However, when comparing our cortisol values obtained from the control group (and ELS) to values derived from large-scale normative paediatric studies, our values lie within one standard deviation (Tollenaar et al. 2010; Törnhage 2002; Tzortzi et al. 2009). Moreover, when comparing the values from our ELS youths specifically to studies with maltreated youths in the same age range (Badanes et al. 2011; Cicchetti and Rogosch 2001b), we obtained results that lay below one standard deviation. In a similar vein, it has been shown that there can be substantial intra-individual variance in cortisol values from day to day, either due to previous day stressors, ultradian spikes or measurement error induced by non-adherence to the sampling protocol (Almeida et al. 2009; Dahlgren et al. 2009). This could potentially limit the generalizability of our findings, because we sampled only on two consecutive days. However, in our sample, measurements for both days were highly correlated and therefore combined to obtain a more stable measure of cortisol production that reflects general diurnal levels.

Another limitation is the unknown genetic makeup of the youths in this study, as it has been suggested that certain genotypes can increase neuroendocrine sensitivity to stress (Bick et al. 2012; Sumner et al. 2014). Specifically, it has been shown that variations in the single nucleotide polymorphisms (SNPs) of the CRHR1 gene (gene for the CRH type 1 receptor) moderated the effect of childhood maltreatment on elevated cortisol responses (Tyrka et al. 2009) and increased levels of depressive symptoms (Heim et al. 2009). Moreover, epigenetic alterations in the glucocorticoid receptor (GR) gene of maltreated individuals and reductions in telomere length (Shalev et al. 2012) have also been identified in animal and human studies (Weaver et al. 2004) and likely contribute to altered stress-reactivity and heightened susceptibility to psychiatric disorders in maltreated and traumatized children. Future studies using interdisciplinary approaches that integrate both the effects of genotype and the epigenetic effects of early adversity into longitudinal investigations will provide vital information.

We assume that the differences between basal cortisol production in ELS youths and controls are likely due to a multitude of adverse factors surrounding the early separation from the caregiver. However, in this study we added evidence to the existing literature by showing that increased psychopathological symptom complexity and hypocortisolism can still be detected in youths with ELS that otherwise seem to have achieved “optimal outcomes” (e.g. good caregiver relationships; school achievements and absence of affective psychopathology).
